# The Development of a Brief but Comprehensive Therapeutic Assessment Protocol for the Screening and Support of Youth in the Community to Address the Youth Mental Health Crisis

**DOI:** 10.3390/brainsci14111134

**Published:** 2024-11-10

**Authors:** Margaret Danielle Weiss, Eleanor Castine Richards, Danta Bien-Aime, Taylor Witkowski, Peyton Williams, Katie E. Holmes, Dharma E. Cortes, Miriam C. Tepper, Philip S. Wang, Rajendra Aldis, Nicholas Carson, Benjamin Le Cook

**Affiliations:** 1 Cambridge Health Alliance, 1493 Cambridge St., Cambridge, MA 02139, USA; erichards@challiance.org (E.C.R.); dbienaime@challiance.org (D.B.-A.); twitkowski@challiance.org (T.W.); pewilliams@challiance.org (P.W.); cholmes@challiance.org (K.E.H.); decortes@challiance.org (D.E.C.); raaldis@challiance.org (R.A.); ncarson@challiance.org (N.C.); bcook@cha.harvard.edu (B.L.C.); 2 Department of Psychiatry, Harvard Medical School, Boston, MA 02215, USA; 3 Department of Psychiatry, Vagelos College of Physicians and Surgeons, Columbia University, New York, NY 10032, USA; miriam.tepper@nyspi.columbia.edu; 4 New York State Psychiatric Institute, New York, NY 10032, USA; 5 Center for Learning Health Systems, Brigham and Women’s Hospital, Boston, MA 02115, USA; pswang@mgb.org; 6 Department of Psychiatry, Harvard University, Boston, MA 02115, USA

**Keywords:** access to treatment, health equity, pandemic, youth mental health, virtual research

## Abstract

Objective: The objective of this study was to explore the acceptability and feasibility of a therapeutic assessment protocol for the Screening and Support of Youth (SASY). SASY provides brief but comprehensive community-based screening and support for diverse youth in the community. Methods: SASY screening evaluates symptoms, functioning and clinical risk. The Kiddie Computerized Adaptive Test was used to evaluate seven different diagnoses and symptom severity. The Weiss Functional Impairment Rating Scale-Self was used to measure functional impairment. Measures were scored according to nationally developed norms. An algorithm was developed to aggregate symptom and function ratings into an overall score for clinical risk. The results are discussed with participants in a motivational interview designed to promote insight, followed by the opportunity for the participant to engage in an online intervention. Protocol changes necessitated by social distancing during the pandemic led to innovative methods including the use of a QR code for recruitment, integration of both online and offline participation, and expansion from in-person recruitment within the schools to virtual engagement with youth throughout the community. The final sample included disproportionately more Black or African American and Hispanic youth as compared to school and community statistics, suggesting that optimization of online and offline methods in research may facilitate the recruitment of diverse populations. Qualitative interviews indicated that the screening and feedback raised youth awareness of their wellbeing and/or distress, its impact on their functioning, and engagement with options for improved wellbeing. Conclusions: The emergence of innovative methods optimizing the advantages of both online and offline methods, developed as a necessity during the pandemic, proved advantageous to the feasibility and acceptability of community-based recruitment of at-risk, minoritized youth.

## 1. Introduction

We describe the development of a therapeutic assessment protocol, Screening and Support for Youth (SASY), to address the youth mental health crisis To provide the background leading to this initiative, this introduction will review the youth mental health crisis (YMHC) itself, the methods required for research into the YMHC, the constraints the pandemic imposed on research in general and on research with youth, and how this led to the innovative methods incorporated into the development of SASY. We conclude that the optimization of both online and in-person methods during the pandemic contributed to our success in recruiting and engaging with minoritized youth. See Graphical Abstract.

### 1.1. The Youth Mental Health Crisis

A 2021 Centers for Disease Control and Prevention (CDC) report drawn from nine nationally representative data sets found that although youth mental health was deteriorating prior to the pandemic, this situation worsened substantially with the onset of the COVID-19 pandemic [1]. Persistent feelings of sadness and hopelessness increased from 36% in 2011 to 57% in 2021. More than 42% of high school students and 60% of female students reported feeling sad or hopeless to such an extent that they could not engage in regular activities [1]. In the 2022 Mental Health Surveillance, 20.9% of youth had a major depressive disorder, 36.7% felt persistently sad, and 18.8% had seriously considered attempting suicide [2].

The American Academy of Child and Adolescent Psychiatry and the American Academy of Pediatrics issued a joint declaration of a national emergency in adolescent mental health [3]. The surgeon general, Vivek Murthy, issued an advisory bringing the youth mental health crisis to national attention, noting “It would be a tragedy if we beat back one public health crisis only to allow another to grow in its place” [4]. The advisory was a call to action to recognize the impact of the youth mental health crisis and assure access to care. Importantly, the advisory’s last recommendation was to “increase timely data collection and research to identify and respond to youth mental health needs more rapidly”, especially the “needs of at-risk youth”, and to “engage directly with young people to understand trends and design effective solutions.” Further, it was recommended that schools “learn how to recognize signs of changes in mental and physical health” [4].

This prompted a Morbidity and Mortality Weekly Report (MMWR) from the Centers for Disease Control (CDC), showing that children living in poverty and minoritized children fare worse than their peers in terms of access to care, risk, and prevalence of mental health disorders, including the evaluation of a wide range of symptoms, the impact of symptoms on functional impairment, suicidality, and analyses of wellbeing with qualitative interviews [5,6]. In October 2021, guidance from the Public Health Informatics Institute suggested future directions including ongoing surveillance and the recognition that “[although] current data sources and measures provide some information on specific disorders and indicators of mental health, the data are not sufficient to provide a comprehensive description of children’s mental health in the United States” [3]. Shim et al. noted that studies attempting to understand the youth mental health crisis must include “surveillance systems [that are] more comprehensive, reporting key elements that can guide transformation in children’s mental health” [6].

### 1.2. The Impact of the Pandemic on Research Methods and Research Equity

During the peak of the pandemic, up to 1100 research trials were stopped per month [7], with significant challenges to data quality [8]. The research studies that remained operational, like ours, had to pivot to teleresearch procedures [9,10]. A narrative review of 43 successful studies demonstrated that the adaptations that were required for success included remote monitoring, online data collection, online interviews, the development of virtual platforms for participant interaction and questionnaire completion, participant compensation, and risk assessment for vulnerable patients [11]. Trials that succeeded during the pandemic did so because they were able to improvise methods for moving from in-person to online procedures [12], which, in turn, required paying greater attention to protecting scientific integrity [13].

We use the term ‘hybrid research’ to refer to optimizing the distinct advantages of both online and offline methods. Research that allows for online participation, sometimes referred to as ‘decentralized’ research, allows participants to engage in research without having to travel to a brick-and-mortar site. An unanticipated consequence of expansion of online methods may be improved recruitment of minoritized participants. Prior to the pandemic in 2020, only 8% of clinical trial participants were Black, 6% were Asian, and 11% were Hispanic [8]. Decentralized approaches place research in the community, which, in turn, directly impacts health equity in research by facilitating the inclusion of populations who would otherwise be unlikely to participate [7,8]. Hybrid research has the potential to bridge barriers when researchers and participants cannot easily be co-located [8], while at the same time supporting the inclusion of subjects who are more likely to engage in person. Improving diversity in research participation has been called an “ethical and scientific imperative” [7,8], and hybrid research may be moving research forward to achieve that imperative.

### 1.3. Hybrid, Brief but Comprehensive Methods in Youth Mental Health Research

Existing epidemiological studies suggest the need for a more in-depth appraisal of a sample of diverse youth [14]. Merikangas et al. [15] identified the failure of current epidemiological methods to address mental disorders that are accompanied by “significant life impairment in individual, educational, or social functioning” as a major gap in our ability to track the emotional and behavioral wellbeing of youth. They argue that in order to accomplish this objective, it is necessary to use internet-based tools that can “bridge the gap between population-based research and interventions at both the societal and individual levels” [15]. To shift the landscape of epidemiology in child psychiatric conditions, Merikangas et al. noted that we must move towards a new model that addresses both mental disorders and “significant life impairments”. They recommend “more comprehensive assessments in smaller well-defined subsets” that “could provide a full portrait of the spectrum of mental health and disorders in the youth population” [15]. Further, they note pervasive multimorbidity with mental disorders, meaning that we need to shift from single diagnosis to “person-based estimates that identify health services needs” [15]. This requires methods that identify multiple diagnoses in any individual, and characterize youth with impairments even in the absence of specific diagnoses. Internet-based tools can accomplish this challenge.

### 1.4. Development of a Scalable, Therapeutic Assessment Protocol

We responded to the need for hybrid, brief, and comprehensive methods in youth mental health research by developing a scalable therapeutic assessment protocol, namely Screening and Support for Youth (SASY). The screening is used to evaluate multimorbidity, functional impairment, and overall risk. SASY directly engages youth in a motivational interview to promote insight and explore options for building coping mechanisms and resilience. As suggested by Merikangas [15], SASY successfully uses online internet tools to bridge the gap between community-level assessment, and social and individual intervention. SASY optimizes online methods with offline in-person methods as needed to promote personal connection. The development of the therapeutic assessment protocol for SASY required innovative methods for recruitment, consent, computerized adaptive assessment of symptoms, diagnoses, and suicidal risk, communication of feedback, data collection, and direct data entry from computerized measures into REDCap [16].

### 1.5. Impact of Hybrid Methods on Recruitment of Minoritized Youth

The burden of in-person participation in research studies within disadvantaged communities may include barriers to travel and increased demands on time. Since online research is asynchronous, youth can participate outside of school hours. Online research does not require schools to provide space for research. On the other hand, flexible inclusion of offline methods may help to engage youth whose participation is motivated by a wish for in-person contact and the connection that grows out of a face-to-face relationship, as well as building community connection through a physical presence in the schools.

Previous research has demonstrated that participatory, decentralized approaches to research can improve ethically responsible research with vulnerable populations who are ‘hard to reach’ or ‘hidden’ [17]. Socially isolated, minoritized, and disadvantaged youth during the pandemic represent just such a vulnerable population who in the past have been unwittingly excluded from research participation [18]. A systematic review described the limited research to date on the challenges of engaging minoritized or disadvantaged participants in community research [18,19]. These challenges are particularly pertinent to working with youth, and even more pertinent to working with minoritized youth, for whom logistical, ethical, and developmental challenges further compound the complexity of engagement [20].

The acceptability and feasibility of SASY in school and community settings demonstrate how innovative research methods can contribute to research on the youth mental health crisis. To our knowledge, this is the first in-depth community study of youth self-reported mental health that combines comprehensive evaluation of seven diagnoses, evaluation of the impact of symptoms on domains of functional impairment, and triage for risk, with a feedback session to promote engagement in a scalable online resilience intervention.

The implementation of the methodological innovations described in this paper together accomplished Merikangas’ description of the need for internet-based tools that address multimorbidity and life impairment in a comprehensive assessment of a subset of the community, with the goal of transforming youth mental health.

## 2. Methods

### 2.1. Study Design

This was an observational, mixed-methods (i.e., qualitative and quantitative) study looking at the feasibility and acceptability of the screening and feedback interventions carried out in diverse communities adjacent to the Boston area. Quantitative data captured recruitment and completion rates of the SASY. Qualitative data gathered in semi-structured interviews provided information about their experiences with the recruitment, screening, and feedback and referral processes with 20 youth and two parents. Youth participating in qualitative interviews were stratified into four groups according to race and language and received a USD 25 incentive for participation. Youth participating in screening received a USD 10 incentive for participation. A wage payment model was used to determine an appropriate incentive for adolescents, based on the time and effort required for participation [21]. This study was approved by the Cambridge Health Alliance IRB.

### 2.2. Recruitment

**Figure 1:**
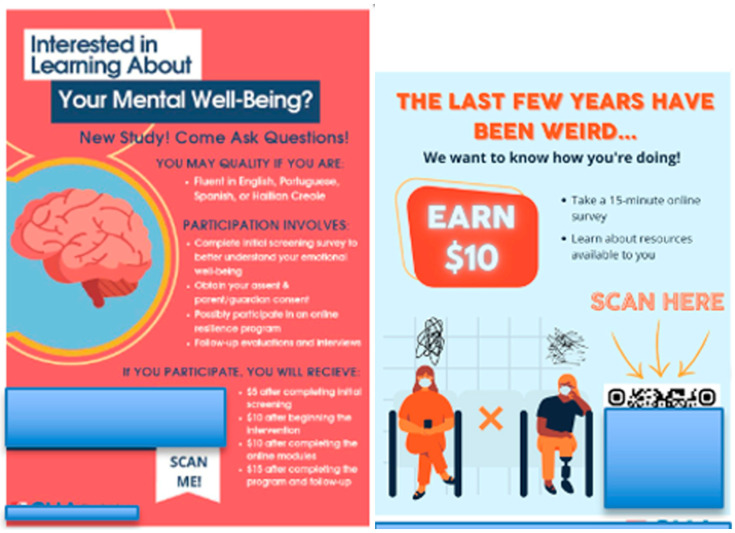
Youth responsive flyers.

Youth were recruited through flyers (Figure 1) with QR codes, word of mouth, tabling at events, and face-to-face contact in the schools. Community advisory boards composed of youth were instrumental in facilitating recruitment strategies and developing flyers. Multiple recruitment strategies were used and tested in an iterative model over time. These strategies included the physical presence of research assistants in the teen health clinics in each school, in sports events, at the library, at school events, at community fairs, and in pediatric offices. Research assistants presented the project directly in classrooms. School staff and nurse practitioners in the clinics worked directly with research staff to alert students who might be interested to the study. Research assistants responded in a timely manner when students reached out responding to the QR code on the flyers that were posted in schools, and in areas adjacent to schools where youth tend to congregate. Word-of-mouth networking was encouraged when students participated and enjoyed the experience of referring their friends.

### 2.3. Description of Screening and Support for Youth (SASY)

The SASY intervention integrates four components, of which the first two are commercially available.

1. Assessment of multimorbidity including depression, anxiety, mania/hypomania, ADHD, oppositional defiant disorder, conduct disorder, substance use, and suicidality.
2. Evaluation of how emotional and behavioral symptoms drive life impairments in family, school learning and behavior, life skills, self-concept, social engagement, and risky activities.
3. Generation of a clinical risk score defined according to an algorithm that integrates the severity of symptoms and functional impairment as compared to national norms developed prior to the pandemic. In this risk score, Tier 1 was considered normal, Tier 2 was considered at risk (i.e., <2 diagnoses and functional impairment less than 1 SD outside the norm), and Tier 3 was considered clinically ill (i.e., two or more diagnoses rated moderate or severe with clinically significant functional impairment > 1.5 SD outside the norm).
4. A motivational feedback session. This brief interview takes place immediately after the screening and provides the participant with feedback on the results, and open-ended questions to better understand their perception of their symptoms and functioning, strengths, wellbeing, and coping strategies. The feedback session allowed youth who expressed distress to use this insight to create an opportunity for engagement in building resilience through their own initiatives or through participation in an online intervention. The maximum total time to complete the entire SASY intervention was thirty minutes.

### 2.4. Participant Disposition Post-Screening

The online intervention is a commercially available program, COPE2Thrive (C2T) (cope2thrive.com), that could be accessed by all participants, irrespective of their clinical Tier. C2T is adapted from an evidence-based in-person CBT intervention that has been shown to improve health behaviors and mood [22,23,24]. The online format version has not previously been tested for effectiveness. Tier 3 participants were given information about potential treatment resources. Given lengthy waitlists, participants awaiting assessment or treatment were eligible to participate in C2T. Overall, 27.9% of Tier 3 participants were already in treatment and were excluded from participation in the online program to avoid any potential conflicts with their current clinical treatment.

### 2.5. Measures

#### 2.5.1. Symptoms and Diagnosis

Psychiatric symptoms and diagnoses were assessed using the Kiddie Computerized Assessment Test from Adaptive Testing Technologies (K-CAT). The K-CAT draws from a bank of 2120 items using an adaptive algorithm to provide a score for each diagnosis as well as risk of suicide [25]. It has been validated against the K-SADS structured clinical diagnostic interview in youth up to age seventeen years for each of the above disorders with AUCs of 0.83 for Generalized Anxiety Disorder (GAD), 0.92 for Major Depressive Disorder (MDD), and 0.996 for suicidal ideation or attempts [26]. Each positive diagnosis was assigned a severity score of mild, moderate, or severe.

#### 2.5.2. Functional Impairment

As part of the assessment of functional impairment, the respondent is asked how emotional or behavior problems over the last month have impacted functional well-being, relative to how they would be functioning if they were asymptomatic. Relative functional impairment is a measure of state that is highly sensitive to change since it will improve or worsen depending on the status of the participant’s mental health [27,28].

Functional impairment was measured by youth report on the Weiss Functional Impairment Scale-Self Report (WFIRS-S). The WFIRS-S has been translated and validated in 22 languages, with each of the domains matching to a distinct factor [27,28]. More recently, a large validation study by Multi-Health Systems of over 3000 participants in the US has generated a manual that includes item- and domain-specific norms, meaning that each domain and the total score can be classified as normal, at risk, or impaired [27].

The WFIRS-S is adaptive in that youth only complete the items that are relevant to them, which are rated from 0 (not at all) to 3 (very much). The domain and total scores are generated using the mean of the relevant items, with scores between 0.8 and 1 (out of a potential total score of 3) being one standard deviation outside the norm, and scores greater than 1 being more than 1.5 standard deviations outside the population mean [28].

#### 2.5.3. Clinical Risk Score

**Figure 2:**
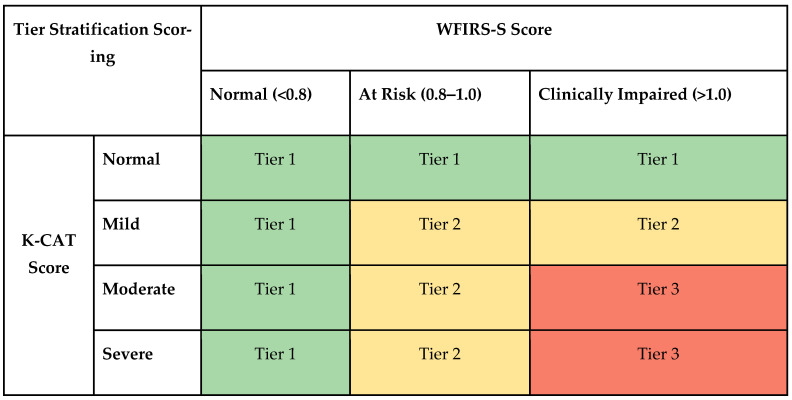
Algorithm for combining symptoms and functioning into a clinical risk score.

An algorithm was developed to integrate the symptom and function scores into a single score, through careful consideration of how symptoms and impairment uniquely contribute to clinical status. Figure 2 illustrates the algorithm. Participants who were functioning in the normal range were considered Tier 1 or ‘normal’, even if they were symptomatic, given that criteria for illness or diagnoses in the DSM requires that symptoms are associated with some degree of functional impairment. Participants who were asymptomatic but described mild functional impairment (up to 1 SD) were also classified as Tier 1 (normal). Participants who had both moderate to severe symptoms and were at risk (between 1 and 1.5 SD) for functional impairment were classified as Tier 2 (at risk). Participants who had mild symptoms but felt those symptoms were causing clinically significant functional impairment were also considered Tier 2.

Participants in Tier 3 were both moderately or severely symptomatic for two or more of the seven diagnoses, and those symptoms had to be causing functional impairment more than 1.5 SD outside the norm. The cut off for Tier 3 represents a particularly robust threshold for clinical severity, given as follows: diagnoses rated as severe, multiple diagnoses, and clinically significant functional impairment. In addition, we created a fourth group classified as ‘High Risk’ based on a rating of severe on the suicide severity scale on the K-CAT. A participant could be “High Risk” regardless of their Tier assignment if they were suicidal on the K-CAT.

#### 2.5.4. Suicide Risk

A common barrier to universal screening for suicide is concern regarding documentation of suicidal risk in the absence of having a procedure in place for an immediate clinical response. This is difficult to operationalize when there is a time lag between when a screening is carried out, when it is interpreted, and when the individual receives an appointment. The SASY includes direct and immediate clinical feedback with the participant, allowing us to respond appropriately in the moment to urgent clinical situations.

A standard operating procedure was developed for triage and a stepped care response to potential suicide risk. The suicide protocol was triggered if a participant received a rating of ‘severe’ on the suicide module of the K-CAT. At that point, the research assistant in the feedback interview administered the Columbia Suicide Severity Rating Scale (C-SSRS) [29]. If the participant scored ‘severe’ on the C-SSRS, the research assistant paged a research clinician who immediately conducted a virtual, typically brief, clinical interview to establish risk and set up an appropriate clinical response as per treatment as usual.

### 2.6. Population

The study population included students attending public high schools in racially, ethnically, and linguistically diverse communities. The majority of participants came from the high schools serving Cambridge (62.1%), Everett (21.5%), and Somerville (11.9%).

### 2.7. Recruitment

Initial recruitment efforts were situated within the teen health centers at each high school. This led to an over-recruitment of females; thus, recruitment was extended to classrooms, school events, sports teams, and then eventually to tabling in local community settings such as libraries or other events. Flyers with QR codes were widely distributed in four languages and were linked to a Google Form in the language in which the QR code was scanned. The Google Form allowed students to input contact information for outreach by the research assistants. Interpreter/translation services were provided for all procedures and documents as needed. All electronic materials and all components of the SASY were available in English and Spanish.

### 2.8. Acceptability and Feasibility

#### 2.8.1. Quantitative Data

Acceptability was assessed by looking at the number of participants who decided to participate in the study. Descriptive statistics were used to describe the age, gender, race, and ethnicity of the study population. This was compared to the demographics of the communities as a whole to evaluate the success of recruitment procedures in enrolling minoritized populations. We compared the percentages of Black or African American, Hispanic, and Asian students from each school to the demographics of the respective schools and communities.

#### 2.8.2. Qualitative Data

We coded verbatim transcripts to assess the feasibility and acceptability of SASY from the perspective of the students. A preliminary codebook using a priori codes based on the interview guide’s domains (acceptability of timing, convenience of the online screening platform, relevance of the questions and usefulness of the screener and feedback). Upon the completion of data coding, a thematic analysis was conducted. This involved categorizing coded interview excerpts under unifying themes to assess the extent to which the intervention was experienced as feasible and acceptable by research participants. The themes resulting from the categorization of coded excerpts are presented below.

### 2.9. Protocol Amendments to Set Up the Decentralized, Hybrid Trial and Optimize Recruitment

**Figure 3:**
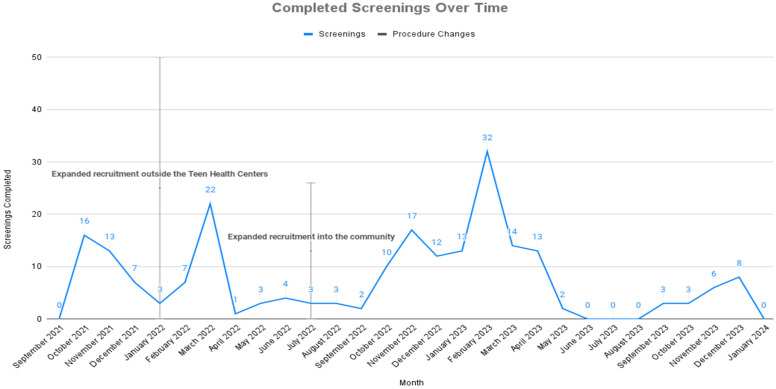
Completed screenings over time.

The United States declared a pandemic emergency in March 2020 and officially declared the end of the pandemic in May 2023. This study ran from November 2020 during school lockdown until May 2024, which was approximately contemporaneous with the pandemic. In March 2022, following the rise in the Omicron variant, a protocol amendment was submitted for the study to run fully virtually if that proved to be necessary. Social distancing procedures and school lockdown required iterative and ongoing demands to revise study methods. Critical protocol amendments are summarized in Table 1. In evaluating the impact of the decentralization and hybridization of online and offline procedures in the project, it should be noted that the date of implementation of the protocol changes often did not go into effect until the following school semester, resulting in the time lag to recruitment illustrated in Figure 3.

Recruitment increased dramatically in February 2022 (when the study was decentralized from the school health centers to the schools as a whole), and then again in October 2022 (when it was decentralized from the schools to the community). Recruitment remained negligible during the summer throughout the study, as would be anticipated in a study of high school students. Decentralization was only possible when we established virtual procedures for the following steps: 1. recruitment (the QR code), 2. waiver for written consent, 3. digital circulation and completion of measures, 4. telehealth feedback, and 5. virtual administration of the suicide protocol.

## 3. Results

A total of 219 participants were recruited, representing a racially and ethnically diverse sample of youth (Table 2). The majority of the youth in the survey were 15–17 years old (67.6%), with a mean age of 16.1; most were female (70.8%) and reported English as their primary language (89.0%).

### 3.1. Recruitment of Diverse Populations

In this study we over-recruited minoritized youth (Asian, Black/African American, Hispanic, and multiracial) participants, relative to the demographics of the community. This is true in comparing the research sample to both the school and community demographics. Overall, 79.9% of the sample came from minority backgrounds, vs. 70.3% of the school populations, although there was a variance in particular ethnicities in particular schools. A comparison of the demographics of study participants in the school vs. the demographics of each community as per the 2020 census data provides us with an indicator of our success in recruiting minorities. Our study sample was 31.5% Black/African American, as compared to 10.6% of the Cambridge population, 5% of the Somerville population, and 13.3% of the Everett population. We recruited 31.5% Hispanic participants as compared to 8.7% of Cambridge, 10.9% of Somerville, and 9.1% of Everett participants. Of particular interest is that 46.6% of the study participants did not speak English at home. Twenty-six parents did not consent, although their children had assented. Our procedures over-recruited minoritized youth relative to the demographics of their particular school, but the demographics of the schools include more minoritized individuals than are found in the community. School-based recruitment in itself is an effective way to engage minoritized youth in research.

### 3.2. Acceptability and Feasibility

#### Qualitative Findings

- Theme 1: Acceptability and ease of screening

During the in-depth qualitative interviews (30–60 min), participants were asked about the acceptability and relative ease of the screening survey. Participants generally agreed that the screening questions were an acceptable and relatively easy way to assess mental health; however, there was some discordance in participants’ feelings about the repetitive nature of some of the questions. One participant noted:

*“…it was pretty smooth to get through. pretty like easy on… I understand why it’s repetitive because it’s trying… different wording changes… which like I understand but I think that… repetitiveness made it a little bit longer. But it didn’t bother me personally, so, I don’t know, I feel like it went pretty smoothly for me.”*

- Theme 2: Screening content and experience

Participants generally reported that the screening questions gave them a unique, new opportunity to think and reflect on their current well-being and personal challenges. They described that the SASY made them think about how they were doing in a new way and how this was affecting them. It prompted reflection on the loneliness they had experienced during the pandemic and how to cope “when you come back from such isolation.” Sample quotations from the qualitative interviews are included in Table 3.

## 4. Discussion

It is feasible and acceptable to run a hybrid trial providing intensive screening and feedback with a diverse population of youth in the community, even within the constraints of a pandemic, school closures, and youth malaise in response to the pandemic [30]. This study faced four particular challenges, any one of which would have been notable; however, combined, they presented a significant barrier to conducting health equity research with diverse youth in the community.

First, community-based research requires recruitment of participants who are not actively seeking care and do not see any personal benefit to participation. Second, adolescents are uniquely difficult to engage and retain [31]. Third, the protocol changes required a switch from an in-person study to a hybrid online/offline study; this required extensive investment of staff time in protocol amendments requiring IRB approval. Fourth, past research has often failed to include adequate representation of diverse populations. It has been suggested that decentralized virtual trials impact the demographics of the population recruited, both to include a more diverse population or to present new or different barriers to recruitment for some populations [7]. Our hypothesis that decentralization and hybrid methods are among the ingredients that led to our success in recruiting minority populations needs to be replicated to test the feasibility of future health equity research with youth.

The method changes described above are consistent with the literature on the impact of the pandemic on research in general during that time period [11,32,33,34]. The studies that were successful in remaining operational were studies that successfully negotiated the pivot to teleresearch procedures [9,10].

The adaptation to a decentralized, hybrid study required 18 IRB protocol amendments to create the iterative adaptations needed to semi-flexibly adapt to shifting real-world circumstances. The innovations which we have described in this pilot have important implications for future studies focused on minoritized youth. These include the use of school-based recruitment in diverse communities, the use of a QR code to recruit participants, comprehensive but rapid computerized adaptive screening of multimorbidity and functional impairment, aggregation of symptom and function scores to generate a tiered risk score, work with a youth community advisory board, and waiving documentation of consent. Direct engagement with youth regarding their perspective on the results of their screening and how they are feeling through feedback sessions and qualitative interviews personalized the findings and increased engagement, both with the study and with the online intervention. Future research is needed to determine which of our procedures was most effective in the recruitment of at-risk underserved youth, and if the SASY procedure has therapeutic value in its own right by providing youth insight into emotional well-being and engagement with intervention.

The method described here accomplishes Merikangas’ call for more comprehensive assessments with smaller well-defined subsets of the population evaluating patient-specific multimorbidity of various symptoms and diagnoses along with their associated life impairments. Only with precision and personalized evaluations can we identify health service needs and the potential impact of scalable interventions that can address the social crisis of youth mental health. The integration of screening data into the three tiers to identify those youth who are either at risk or clinically ill is essential to the development and implementation of best practice for selecting between universal, targeted, and clinical interventions [35,36].

An important and unanticipated dividend of the hybrid teleresearch methods we have described was our success in the recruitment of minoritized youth. We over-recruited Black or minoritized youth across all communities, despite the fact that these populations are historically underrepresented in research samples. Given that the youth mental health crisis is believed to have had a differential deleterious effect on minority youth [37], and that minority youth are significantly underrepresented in behavioral health care and research [38], this has important implications for engagement of the youth most in need of support in our schools and least likely to receive it.

Although we cannot say with certainty which components of SASY were attractive to minoritized youth, we have several hypotheses that can be tested in future studies. First, the racial, ethnic, and linguistic demographics of the research staff were concordant with the population we wished to study, including young adult individuals who were Black, Hispanic, and/or who spoke Haitian Creole and Spanish. Second, our adjustments to study procedures greatly mitigated the burden of participation while simultaneously providing an opportunity for participants to access screening, feedback, and the online intervention they could not otherwise have received except at considerable out-of-pocket costs. Participants could consent, complete measures, and receive feedback without missing school and without their parents missing work. At the same time, those who preferred in-person contact had that option available to them. Extensive support and interpreter services were available to assist parents who did not speak English or were not digitally literate. This was critical in a study in which almost half the participants came from families in which the parents do not speak English, and it is essential to research in the assessment of the mental health of recent refugees or immigrants. Although we over-recruited minoritized populations relative to school demographics, recruitment within schools in which these minorities were themselves overrepresented as compared to the wider community was critical to our success.

There are two outcomes of our testing the feasibility and acceptability of SASY that we believe to have important potential clinical implications. The feedback interview promoted insight by youth into their own distress, and how that distress was impacting their well-being. This, in turn, prompted engagement with community-based or digital opportunities for support. Given that minoritized youth are under-represented in mental health services [39], this has implications for decreasing inequities in behavioral health treatment. Multi-tiered screening for suicide at the community level using the K-CAT, on an individual level using the C-SSRS, and then at the clinical level once risk had been established was an efficient and effective way of screening for suicide while mitigating potential risk. Future studies will look at the sensitivity, specificity, and correlation between population, individual, and clinical screening for suicidal risk.

### Limitations

There are limitations to consider in this study. The K-CAT does not capture several important conditions, although participants with these diagnoses are considered as being in Tier 3 (clinically ill) if they are functionally impaired as a result of their symptoms. Our definition of Tier 3 was more robust than what would be considered the cut-off for diagnosis in most structured interviews or in clinical settings. This means that the results of the study are conservative, leaning towards an underestimate of psychopathology as compared to the methodology of most epidemiological surveys. We failed to recruit representative samples of students who did not speak English, although almost half the sample came from homes where the first language is not English. This was despite ensuring that all study materials were available in the four most common languages in the relevant communities, as well as ensuring access to interpreters. The use of commercially available, but well-validated and normed, tools means that the implementation of SASY requires access to funding.

## 5. Conclusions

The development of SASY allowed us to operationalize Shim’s demand to create “surveillance systems that are more comprehensive, reporting key elements that can guide transformation in children’s mental health” [6]. This study demonstrates that it is feasible and acceptable to address the Surgeon General’s call for “timely data collection and research to identify and respond to youth mental health needs more rapidly”, the “needs of at-risk youth”, and to “engage directly with young people to understand trends and design effective solutions” [4].

## Figures and Tables

**Table 1:** Iterative adaptations of the protocol.

Date	Iterative Changes
Iterative changes throughout the study	Improved youth appeal of flyers.
	Introduced and improved the QR code and URL link to widen recruitment.
February 2021	Set up high-risk protocol to manage suicidal youth and added the C-SSRS to assess risk for youth with a rating of severe on the suicide module of the K-CAT.
January 2022	Expanded recruitment outside the teen center walls to the school as a whole.
March 2022	Increased screening incentive from USD 5 to USD 10.
	Established procedures to be able to screen virtually as well as in person.
July 2022	Expanded recruitment to the community. Obtained a waiver to allow verbal consent.
August 2022	Adapted the motivational feedback session to emphasize strengths as well as challenges.
August 2023	Offer online C2T program to Tier 1 if interested.

**Table 2:** Demographic characteristics.

Characteristics	Category	Count (n = 219)	Percentage (%)
Age (years)	12–14	29	13.2%
	15–17	148	67.6%
	18–21	39	17.8%
	22–24	3	1.4%
Gender	Female	155	70.8%
	Male	48	21.9%
	Non-Binary	9	4.1%
	Prefer not to answer	7	3.2%
Race *	White	85	38.8%
	Black/African American	69	31.5%
	Hispanic	69	31.5%
	Asian	43	19.6%
	Other	16	7.3%
Ethnicity	American	106	48.4%
	African American	34	15.5%
	Asian	35	16.0%
	Brazilian/Portuguese	27	12.3%
	Haitian	20	9.1%
	Hispanic	52	23.7%
	Jamaican	8	3.7%
	Other/Unknown	76	34.7%
Primary language	English	195	89.0%
	Spanish	10	4.6%
	Portuguese	4	1.8%
	Haitian Creole	3	1.4%
	Other	7	3.2%
Language spoken at home	English	117	53.4%
	Spanish	29	13.2%
	Portuguese	17	7.8%
	Haitian Creole	13	5.9%
	Other	43	19.6%
Are you currently receiving professional help for emotional problems?	Yes	61	27.9%
	No	158	72.2%

* Participants were able to select up to three races, and the percentages reflect the percentage of the sample that identified as the respective race.

**Table 3:** Samples from the qualitative interviews.

Participant	Quotes
Youth 1	“It definitely made me think about everything a lot more than I usually do. …I just made me really give myself… a reality check… like what am I actually feeling? How am I actually doing? Which I don’t usually do.”
Youth 2	“I think the surveys, like, forced me to ask myself questions. …the survey just had, like, a reflective aspect, like, on how stressed have I actually been in the last 30 days and then thinking about that. So, I think I did probably attribute some of that to taking those surveys. And like I think the fact that it forced me to check in with myself over a period of time and see that growth or in that case it was just continually getting more stressed because at that point it was just like building up. …I learned from the survey that I was feeling a lot of stress, which was affecting the quality of my relationships. And so, I decided to do something about my workload.”
Youth 3	“I don’t know how much I got out of this survey. I think the survey just made me realize, like, how much I had been like struggling with anxiety overall because I don’t think that I really like took a second to realize like how much I was worrying or feeling angry at people or why I was even feeling that way, it definitely began like my journey into realizing and just having sort of an awakening on how much I’ve been feeling that way. …I think this survey could definitely make anyone realize how isolated we were as a community during COVID. Um, because when you come back from such an isolation like that after like a year of just being on the computer and doing school online, it can make you realize, like how different your life is or how much anxiety it caused, and how now, even in your family life, what’s going on, your friends, like, things like that.”

## Data Availability

The data in this publication can be shared upon request to the corresponding author due to the data is restricted from open access because demographic data includes information that could create potential privacy concerns.
